# Autoantibodies against nephrin and podocin are associated with disease severity and steroid dependence in adult-onset nephrotic syndrome

**DOI:** 10.1038/s41598-026-43612-7

**Published:** 2026-03-16

**Authors:** Norifumi Hayashi, Ryoko Akai, Yu Kagaya, Keiji Fujimoto, Takao Iwawaki, Hitoshi Yokoyama, Kengo Furuichi

**Affiliations:** 1 Department of Nephrology, School of Medicine, Kanazawa Medical University, Uchinada, Ishikawa, Japan; 2 Division of Cell Medicine, Department of Life Science, Medical Research Institute, Kanazawa Medical University, 1-1 Daigaku, Uchinada, Ishikawa 920-0293 Japan

**Keywords:** Biomarkers, Diseases, Nephrology

## Abstract

Nephrotic syndrome is a glomerular disorder characterized by heavy proteinuria and hypoalbuminemia. Autoantibodies against slit diaphragm proteins such as nephrin and podocin have been identified in subsets of patients, but their prevalence and clinical significance in adult-onset disease are not fully understood. We retrospectively studied 114 adults with biopsy-proven nephrotic syndrome, including minimal change nephrotic syndrome, focal segmental glomerulosclerosis, and phospholipase A2 receptor- and neural epidermal growth factor-like 1-associated membranous nephropathy. Serum anti-nephrin and anti-podocin autoantibodies were quantified using enzyme-linked immunosorbent assays.

Anti-nephrin autoantibodies were detected predominantly in minimal change nephrotic syndrome (38.2%) and less frequently in focal segmental glomerulosclerosis (14.3%), but were rare in membranous nephropathy. In contrast, anti-podocin autoantibodies were observed across disease types and were most frequent in neural epidermal growth factor-like 1-associated membranous nephropathy (30.8%). Autoantibody-positive patients presented with more severe nephrotic syndrome, and antibody positivity was associated with a higher incidence of steroid-dependent nephrotic syndrome in exploratory multivariable analyses. In patients with paired samples, autoantibody titers decreased or disappeared in remission.

Notably, anti-nephrin antibodies were preferentially associated with MCNS, whereas anti-podocin antibodies were detected across disease entities and may reflect the extent of podocyte injury rather than disease specificity. Moreover, combined assessment of anti-nephrin and anti-podocin antibody status provided improved stratification of baseline disease severity and relapse-prone disease compared with anti-nephrin antibody status alone.

**Supplementary Information:**

The online version contains supplementary material available at 10.1038/s41598-026-43612-7.

## Introduction

Nephrotic syndrome (NS) is a glomerular disorder characterized by heavy proteinuria and hypoalbuminemia. In adults, particularly in younger individuals, minimal change nephrotic syndrome (MCNS) and focal segmental glomerulosclerosis (FSGS) are two major pathological entities ^1^. MCNS is characterized by minimal changes on light microscopy and diffuse podocyte foot process effacement on electron microscopy. It typically responds well to corticosteroid therapy and has a favorable prognosis. In contrast, FSGS shows segmental sclerosis of glomeruli and often leads to progressive renal dysfunction with a poorer response to immunosuppressive treatment ^2^.

The etiology of MCNS and FSGS is heterogeneous, encompassing both genetic and acquired causes. Mutations in genes encoding podocyte or slit diaphragm proteins account for a significant proportion of pediatric cases and some adult-onset cases ^3^. However, the majority of adult patients present without identifiable monogenic causes ^4^, suggesting that immune-mediated mechanisms play a central role. Historically, MCNS and FSGS were considered to result from systemic abnormalities of T lymphocytes, leading to the production of cytokines that directly or indirectly impair glomerular function ^5^. However, a humanized mouse study demonstrated that albuminuria could be transferred by CD34⁺ hematopoietic progenitor cells but not by mature T cells, indicating that pathogenic immune mechanisms extend beyond the classic T-cell–only hypothesis ^6^. Subsequently, observations such as the rapid recurrence of massive proteinuria with podocyte foot process effacement after kidney transplantation ^7^, as well as the favorable response to B-cell depletion therapy, have suggested the presence of circulating pathogenic factors ^8^.

More recently, autoantibodies against nephrin, a key slit diaphragm protein in podocytes, have been identified in pediatric and adult patients with MCNS ^9^. In addition, novel autoantibodies targeting other slit diaphragm components, such as podocin (NPHS2) and Neph1 (KIRREL1), have also been reported ^10,11^. However, the biological significance and clinical implications of these antibodies, particularly in adult-onset nephrotic syndrome, remain incompletely understood. The aim of the present study is to investigate the presence of circulating anti-nephrin and anti-podocin antibodies in adult patients with NS, including MCNS, FSGS, and membranous nephropathy (MN), and to evaluate their associations with clinical characteristics and outcomes.

## Materials and methods

### Patients

We retrospectively enrolled 114 Japanese adult-onset (≥ 18 years) nephrotic syndrome patients with MCNS (n = 47), FSGS (n = 14) and MN (n = 53) who were admitted to Kanazawa Medical University Hospital. Diagnosis was confirmed by kidney biopsy in all cases. MN was subclassified as M-type phospholipase A2 receptor (PLA2R)-associated MN or neural epidermal growth factor-like 1 (NELL1)-associated MN according to renal biopsy immunostaining for PLA2R and NELL1, as previously described ^12^. For patients with FSGS, we additionally reviewed clinical records for potential secondary/adaptive causes and recorded whether targeted genetic testing for monogenic nephrotic syndrome was performed. The patients were treated non-randomly, depending on the judgment of the doctor in charge of each case (Supplementary Table S1). For analyses of treatment response (remission, relapse, and steroid-dependence), we included only patients who received immunosuppressive therapy. The protocol of this study was approved by the Clinical Study Ethics Review Board of Kanazawa Medical University (approval No.C103). Prior to the study, verbal/written informed consent was obtained from all patients. This study was conducted according to the principles of the Declaration of Helsinki.

### Samples

Blood samples were collected from patients prior to the administration of immunosuppressants and from healthy controls (n = 40; median [IQR] age, 35.0 [29.8–42.0] years; 70% male). Serum was immediately separated by centrifugation at 3000 × g for 15 min, aliquoted, and stored at − 80 °C until analysis to minimize freeze–thaw cycles.

### ELISA

Serum autoantibodies were measured using an enzyme-linked immunosorbent assay (ELISA) based on previously published protocols with minor modifications. Recombinant human Nephrin (Sino Biological, #17,757-H08H) and Podocin (R&D Systems, #9287-PO) were coated onto ELISA plates at 100 ng/well in coating buffer (BioLegend, #421,701) and incubated overnight at 4 °C. Following washing, plates were blocked with 5% skim milk in PBST for 1 h at room temperature (RT). Patient serum samples were diluted 1:200 in 5% skim milk in PBST and incubated for 1 h at RT. After washing, plates were incubated with biotin-conjugated goat anti-human IgG Fc antibody (Invitrogen, #A18827; 1:1000 dilution) for 1 h at RT. After a subsequent wash, plates were incubated with horseradish peroxidase (HRP)-conjugated avidin (BioLegend, #405,103; 1:1000 dilution) for 30 min at RT. Plates were then washed again and developed using tetramethylbenzidine (TMB) substrate (BioLegend, #421,101) for 15 min, followed by the addition of stop solution (BioLegend, #423,001). Absorbance was measured at 450 nm. Wells without recombinant protein coating were used as background controls, and their optical density (OD) values were subtracted from the corresponding antigen-coated wells for each sample. For generation of standard curves, a primary sheep anti-human nephrin polyclonal antibody (R&D, #AF4269) and rabbit anti-human NPHS2 polyclonal antibody (proteintech #20,384–1-AP) were serially diluted in twofold steps, followed by incubation with a biotin-conjugated donkey anti-sheep or goat anti-rabbit IgG secondary antibody (Invitrogen, #A16052 and #A16114; 1:2000 dilution). The cutoff value for anti-nephrin and anti-podocin antibodies positivity was determined as the maximum values observed in healthy controls to minimize false positivity. All samples were measured in duplicate. Assay quality control and reproducibility are summarized in Supplementary Table S2.

### Clinical end-point definitions

Clinical status was evaluated using the urine protein-to-creatinine ratio (uPCR) and remission categories defined by the Japanese Society of Nephrology ^13^ and KDIGO guidelines ^1^.

#### Japanese society of nephrology (JSN) categories

- Incomplete remission type 2 (JSN-ICR2): uPCR 1.0–3.5 g/gCr with serum albumin > 3.0 g/dL.
- Incomplete remission type 1 (JSN-ICR1): uPCR 0.3–1.0 g/gCr with serum albumin > 3.0 g/dL.
- Complete remission (JSN-CR): uPCR < 0.3 g/gCr with normal serum albumin levels.

#### KDIGO criteria

- Partial remission (KDIGO-PR): uPCR 0.3–3.5 g/gCr with > 50% reduction from baseline.
- Complete remission (KDIGO-CR): uPCR < 0.3 g/gCr with stable serum creatinine and serum albumin > 3.5 g/dL.

#### Other definitions

Relapse: recurrence of proteinuria ≥ 1.0 g/gCr on ≥ 2 consecutive visits after complete remission.

Steroid-dependent nephrotic syndrome (SDNS): two or more relapses occurring during steroid tapering or within 14 days after discontinuation. The estimated glomerular filtration rate (eGFR) was calculated using the equation recommended by the Japanese Society of Nephrology: eGFR (mL/min/1.73 m^2^) = 194 × (serum creatinine)⁻^1^·⁰⁹^4^ × (age)⁻⁰·^2^⁸⁷, multiplied by 0.739 for women.

### Statistical analysis

Continuous variables were summarized as medians with interquartile ranges (25th–75th percentile), and categorical variables were expressed as counts and percentages. Differences in the prevalence of antibody positivity among disease groups were first assessed using Pearson’s chi-square test. When a significant overall difference was observed, adjusted residual analysis was performed to identify groups contributing to the significance. Differences between two groups were assessed using the Mann–Whitney U test, and proportions were compared using Fisher’s exact test. The Kaplan–Meier method was applied to evaluate clinical outcomes, and differences between groups were assessed using the log-rank test. Multivariable logistic regression analysis was performed to identify independent predictors of SDNS after adjustment for age, gender, serum albumin, serum creatinine, and urinary protein (uPCR). Serum anti-nephrin and anti-podocin antibody titers in paired samples (nephrotic vs. remission phases) were compared using the paired Wilcoxon signed-rank test. Exploratory receiver operating characteristic (ROC) analyses were performed to assess the discriminatory ability of baseline anti-nephrin antibody titers for SDNS, and the area under the curve (AUC) was calculated. A two-sided p value < 0.05 was considered statistically significant.

## Results

### Prevalence of anti-nephrin and anti-podocin antibodies among patients

**Fig. 1:**
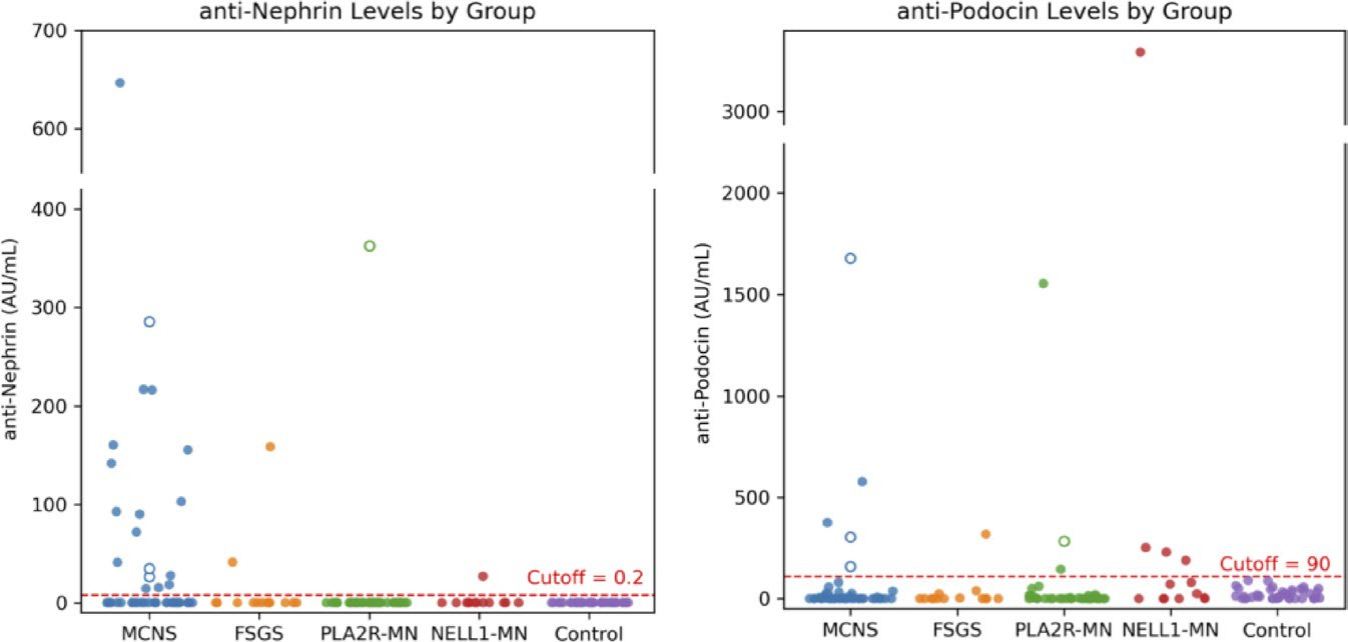
Serum anti-nephrin and anti-podocin antibody levels among glomerular disease groups. Scatter plots show the distribution of anti-nephrin (left) and anti-podocin (right) antibody levels in patients with MCNS, FSGS, PLA2R-MN, NELL1-MN, and controls. Horizontal dashed red lines indicate the ELISA cutoff values (0.2 AU/mL for anti-nephrin and 90 AU/mL for anti-podocin), determined as the upper limit of the control group. Patients double-positive for both antibodies are indicated by open circles of the same color. Anti-nephrin antibodies were predominantly detected in MCNS and FSGS, whereas anti-podocin antibodies were more frequently observed in NELL1-MN.

As shown in Fig. 1, serum levels of anti-nephrin and anti-podocin antibodies varied widely among glomerular disease types. Anti-nephrin antibodies were predominantly detected in patients with MCNS and, to a lesser extent, in those with FSGS, whereas antibody levels in PLA2R- and NELL1-associated MN were generally low. In contrast, anti-podocin antibodies were detected across several disease types, with particularly high titers observed in a subset of patients with NELL1-associated MN.

Using cutoffs defined by the maximum values observed in healthy controls (anti-nephrin ≥ 0.2 AU/mL; anti-podocin ≥ 90 AU/mL), antibody positivity was determined for subsequent analyses. The prevalence of antibody positivity in each disease group is summarized in Table 1. The prevalence of anti-nephrin antibody positivity differed significantly among disease groups (χ^2^ = 19.49, df = 3, *p* < 0.001). Adjusted residual analysis revealed that positivity was significantly higher in MCNS and lower in PLA2R-associated MN. In contrast, the prevalence of anti-podocin antibodies did not differ significantly across diseases (χ^2^ = 5.70, df = 3, *p* = 0.12). Double positivity for both antibodies was observed in a few cases of MCNS (6.4%) and PLA2R-MN (2.5%).

**Table1:** Prevalence of anti-nephrin and anti-podocin antibodies among glomerular diseases.

Disease	n	Nephrin only ( +)	Podocin only ( +)	Double positive	Negative
MCNS	47	15 (31.9%)	2 (4.3%)	3 (6.4%)	27 (57.4%)
FSGS	14	2 (14.3%)	1 (7.1%)	0 (0%)	11 (78.6%)
PLA2R-MN	40	0 (0%)	2 (5.0%)	1 (2.5%)	37 (92.5%)
NELL1-MN	13	1 (7.7%)	4 (30.8%)	0 (0%)	8 (61.5%)
Control	40	0 (0%)	0 (0%)	0 (0%)	40 (100%)

Values are number (%). Each column (nephrin only, podocin only, double positive, negative) represents mutually exclusive categories. Overall χ^2^ test: Nephrin *p* < 0.001, Podocin *p* = 0.12. Control group excluded from statistical analysis (used for cutoff determination).

## Clinical characteristics according to anti-nephrin and anti-podocin antibody status

### Baseline characteristics in MCNS and FSGS by antibody positivity

Patients with MCNS and FSGS were divided into two groups according to the presence of anti-nephrin and/or anti-podocin antibodies (antibody-positive vs. antibody-negative), and their clinical characteristics and outcomes were compared (Table 2). Among the 61 patients analyzed, 23 (37.7%) were antibody-positive. The antibody-positive group included a significantly higher proportion of males compared with the antibody-negative group (69.6% vs. 34.2%, *p* = 0.009). Serum creatinine levels were higher in the antibody-positive group (median 1.00 [0.93–1.54] mg/dL) than in the negative group (0.89 [0.64–1.11] mg/dL, *p* = 0.049), while eGFR showed a tendency toward lower values without statistical significance (*p* = 0.082). Urinary protein excretion was higher in antibody-positive patients (median uPCR 12.67 [10.02–17.68] g/gCr vs. 10.26 [6.14–13.94] g/gCr, *p* = 0.044).

**Table 2:** Baseline clinical characteristics of patients with MCNS and FSGS comparing antibody-positive (anti-nephrin and/or anti-podocin) and antibody-negative groups.

	Total (n = 61)	Positive (n = 23)	Negative (n = 38)	*p* value
Male/Female	29 (47.5%) / 32 (52.5%)	16 (69.6%) / 7 (30.4%)	13 (34.2%) / 25 (65.8%)	0.009*
Age (years)	58.00 [34.00–70.00]	63.00 [42.50–73.50]	47.50 [31.50–69.50]	0.185
anti-Nephrin titer (AU/mL)	0.00 [0.00–27.74]	71.99 [22.41–157.08]	0.00 [0.00–0.00]	< 0.001*
anti-Podocin titer (AU/mL)	0.00 [0.00–10.22]	23.06 [0.54–119.78]	0.00 [0.00–0.00]	< 0.001*
Cre (mg/dL)	0.99 [0.73–1.22]	1.00 [0.93–1.54]	0.89 [0.64–1.11]	0.049*
eGFR (mL/min/1.73 m^2^)	62.60 [40.90–79.20]	59.70 [36.20–68.70]	66.10 [45.55–84.50]	0.082
TP (g/dL)	4.80 [4.40–5.70]	4.80 [4.40–5.45]	5.00 [4.40–5.75]	0.976
Alb (g/dL)	1.90 [1.30–2.20]	1.60 [1.35–2.00]	2.00 [1.22–2.20]	0.336
IgG (mg/dL)	762.00 [539.00–1019.00]	818.00 [626.50–1026.50]	696.50 [505.25–1014.75]	0.835
IgE (IU/mL)	357.50 [81.42–1663.25]	292.50 [107.50–870.50]	504.50 [81.42–1663.25]	0.827
CH50 (U/mL)	53.40 [48.25–59.18]	54.85 [51.90–60.00]	52.70 [42.80–57.50]	0.131
C3 (mg/dL)	130.00 [114.00–151.00]	142.00 [119.50–157.25]	129.00 [114.00–149.00]	0.121
C4 (mg/dL)	36.00 [29.00–45.00]	39.00 [32.00–48.00]	33.00 [29.00–42.00]	0.113
ANA positive / negative	20 (33.3%) / 40 (66.7%)	8 (34.8%) / 15 (65.2%)	12 (32.4%) / 25 (67.6%)	1.000
uPCR (g/gCr)	11.28 [7.72–14.60]	12.67 [10.02–17.68]	10.26 [6.14–13.94]	0.044*
Selectivity Index	0.13 [0.09–0.19]	0.12 [0.10–0.23]	0.14 [0.08–0.18]	0.843
Urinary β2MG (µg/L)	282.00 [94.00–646.00]	265.50 [113.25–535.50]	282.00 [111.00–752.00]	0.860

Antibody positivity was defined as anti-nephrin and/or anti-podocin antibody positivity.

Values are number (%) and median [IQR, interquartile range].

Abbreviations: Cre, serum creatinine; eGFR, estimated glomerular filtration rate; TP, total protein; Alb, serum albumin; ANA, antinuclear antibody; uPCR, urine protein-to-creatinine ratio; β2MG, urinary β2-microglobulin.

### Analyses stratified by anti-nephrin antibody status

To address the distinct biological implications of anti-nephrin and anti-podocin antibodies, we additionally performed an analysis stratified by anti-nephrin antibody status, excluding patients who were positive only for anti-podocin antibodies (MCNS, n = 2; FSGS, n = 1). In this analysis, baseline clinical characteristics were compared between anti-nephrin antibody–positive and anti-nephrin antibody–negative patients with MCNS and FSGS. The results were largely consistent with those of the broader antibody-positive versus antibody-negative analysis and are summarized in Supplementary Table S5.

### Clinical characteristics of patients positive only for anti-podocin antibodies

Among the 114 patients with nephrotic syndrome, 9 were positive only for anti-podocin antibodies. To further characterize this subgroup, we compared baseline clinical characteristics between anti-podocin–positive and anti-podocin–negative patients among those who were negative for anti-nephrin antibodies (n = 92). As shown in Supplementary Table S6 anti-podocin–positive patients did not exhibit greater nephrotic severity at baseline, with comparable serum albumin levels and proteinuria to anti-podocin–negative patients. Anti-podocin positivity was more frequently observed in male patients and was associated with a trend toward higher serum creatinine levels, whereas eGFR and other clinical parameters did not differ significantly between the two groups.

### Antibody status and treatment response in MCNS and FSGS

Two of 47 patients with MCNS and four of 14 patients with FSGS were managed conservatively without immunosuppressive therapy and were excluded from treatment-response analyses; therefore, treatment-response analyses included 45 patients with MCNS and 10 with FSGS.　Kaplan–Meier analyses were performed to compare the antibody-positive and antibody-negative groups for each clinical outcome, including JSN-ICR1/2, JSN-CR, KDIGO-PR/CR, and relapse-free period after remission. No significant differences were observed between the two groups in any of these outcomes (all log-rank p > 0.05) (Supplemental Fig. S1A). When patients positive only for anti-podocin antibodies were excluded (n = 3), stratification by anti-nephrin antibody positivity yielded consistent Kaplan–Meier results (Supplementary Figure S1B). Remission rates were uniformly high across antibody profiles (Supplementary Table S7).　Among the patients who were followed for more than 2 years (n = 16 in the antibody-positive group and n = 26 in the antibody-negative group), however, the antibody-positive group showed a tendency toward a higher incidence of SDNS (75.0% vs. 42.3%, *p* = 0.057). In multivariable logistic regression analysis adjusting for age, gender, serum albumin, serum creatinine, and urinary protein (uPCR), antibody positivity remained an independent predictor of SDNS (Model A: adjusted OR = 9.88, 95% CI 1.38–70.78, *p* = 0.023, Table 3). When antibody positivity was redefined as anti-nephrin antibody positivity alone after excluding patients positive only for anti-podocin antibodies, anti-nephrin antibody positivity also remained independently associated with SDNS (Model B: adjusted OR = 9.31, 95% CI 1.22–70.98, *p* = 0.031, Table 3).

**Table 3:** Multivariable logistic regression analysis for predictors of SDNS development.

	Model A adjusted OR (95%CI)	*p* value	Model B adjusted OR (95%CI)	*p* value
Age (years)	1.05 (1.00–1.11)	0.052	1.05 (1.00–1.11)	0.054
Gender (Male)	0.23 (0.04–1.23)	0.087	0.16 (0.03–0.97)	0.046*
Serum creatinine (mg/dL)	0.62 (0.19–2.06)	0.439	0.67 (0.20–2.26)	0.520
Serum albumin (g/dL)	0.26 (0.05–1.36)	0.110	0.21 (0.04–1.13)	0.068
Urinary protein (uPCR, g/gCr)	0.90 (0.77–1.05)	0.164	0.86 (0.72–1.02)	0.090
Any antibody positive	9.88 (1.38–70.78)	0.023*	_	_
Anti-nephrin antibody positive	_	_	9.31 (1.22–70.98)	0.031*

Multivariable logistic regression analyses for SDNS were restricted to patients with ≥ 2 years of follow-up. The analyzed sample included 42 patients, of whom 23 developed SDNS.

Model A: “Any antibody positive” was defined as anti-nephrin (≥ 0.2) and/or anti-podocin (≥ 90) positivity. This model included 16 antibody-positive patients (12 SDNS events) and 26 antibody-negative patients (11 SDNS events).

Model B: “Anti-nephrin antibody positive” was defined as anti-nephrin titer ≥ 0.2; cases positive only for anti-podocin antibodies (anti-nephrin < 0.2 and anti-podocin ≥ 90) were excluded. This model included 14 anti-nephrin–positive patients (10 SDNS events) and 26 anti-nephrin–negative patients (11 SDNS events).

Values are adjusted odds ratios (OR) with 95% confidence intervals (CI).

Abbreviations: SDNS, steroid-dependent nephrotic syndrome; uPCR, urine protein-to-creatinine ratio; OR, odds ratios; CI, confidence interval.

An exploratory ROC analysis was performed to assess the ability of baseline anti-nephrin antibody titers to predict SDNS, showing modest discriminatory performance (AUC, 0.63; 95% CI, 0.48–0.78) (Supplementary Figure S2). When patients were stratified by combined anti-nephrin and anti-podocin antibody status, SDNS occurred not only in anti-nephrin–positive patients but also in anti-nephrin–negative/anti-podocin–positive patients, all of whom developed SDNS. Patients positive for both antibodies showed the highest incidence of SDNS, whereas the lowest incidence was observed in double-negative patients (Supplementary Table S7).

### Anti-nephrin and anti-podocin antibodies after remission

**Fig. 2:**
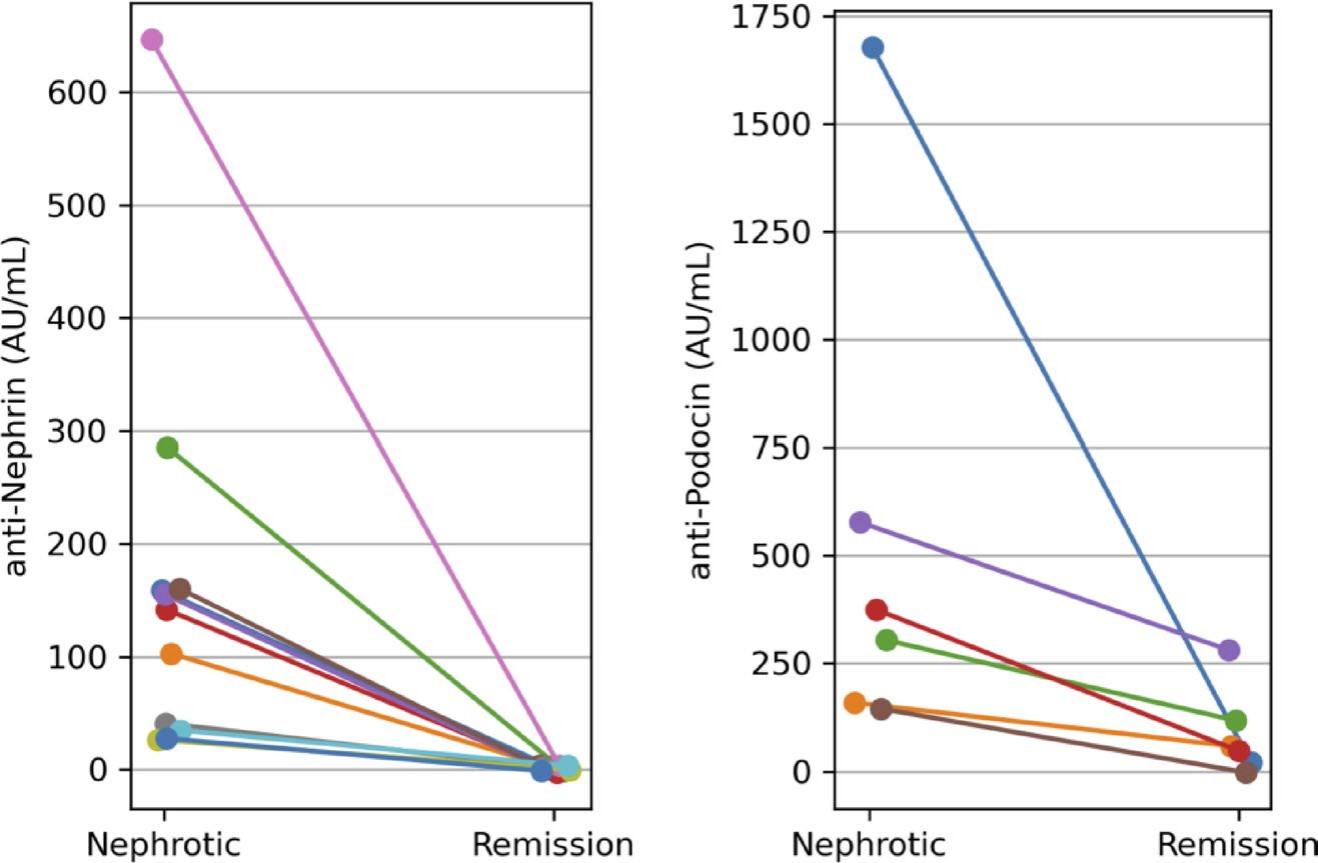
Changes in serum anti-nephrin and anti-podocin antibody titers before and after remission. Paired serum samples obtained during the nephrotic and remission phases were analyzed for anti-nephrin (left) and anti-podocin (right) antibody titers. Each line represents an individual patient. Anti-nephrin antibody titers (n = 11) became undetectable in all remission-phase sera (*p* < 0.001, paired Wilcoxon signed-rank test). Anti-podocin antibody titers (*n* = 6) also decreased significantly after remission (*p* = 0.031).

Among 11 patients with paired serum samples, anti-nephrin antibody titers became undetectable in all remission-phase sera (*p* < 0.001). Anti-podocin antibody titers were available in 6 patients and also decreased significantly after remission (*p* = 0.031), although low-level positivity persisted in 2 cases (Fig. 2).

## Discussion

In this study, we demonstrated that autoantibodies against nephrin and podocin are detectable in a subset of adult patients with nephrotic syndrome. Anti-nephrin antibodies were most frequently observed in MCNS, consistent with recent reports in both pediatric and adult cohorts. Antibodies against nephrin in patients with MCNS were first reported by Watts et al. in 2022 ^9^. Subsequent studies have shown that anti-nephrin antibodies are detected in approximately 30–40% of adults with MCNS, about 10% of patients with FSGS, 40–50% of children with idiopathic NS, and recurrent FSGS after renal transplantation ^9,14–16^. Our findings therefore reinforce the emerging concept that immune-mediated mechanisms involving anti-nephrin antibodies contribute to podocyte injury in a substantial proportion of adult-onset MCNS.

In contrast, anti-podocin antibodies were detected across multiple disease categories, including NELL1-associated MN. Nephrin and podocin are key slit-diaphragm proteins, yet they differ markedly in structure and antigen exposure. Nephrin is a type I transmembrane protein with a large extracellular domain ^17^ and is therefore a plausible target for circulating autoantibodies. Podocin, in contrast, is a membrane-associated scaffolding protein with minimal extracellular exposure ^18^. This raises the question of how anti-podocin antibodies emerge. Notably, in our cohort, anti-podocin antibodies were observed in diverse disease contexts and were particularly frequent in NELL1-associated MN, suggesting that they may arise secondary to podocyte injury and antigen exposure rather than as primary pathogenic drivers. Taken together, these patterns suggest that anti-nephrin antibodies may represent a more disease-enriched autoimmune process, whereas anti-podocin antibodies may reflect secondary antigen exposure accompanying podocyte injury.

We next explored the clinical relevance of these antibodies in MCNS and FSGS. At presentation, patients with anti-nephrin and/or anti-podocin antibodies exhibited greater proteinuria and higher serum creatinine levels than antibody-negative patients, suggesting an association with more severe disease. However, remission rates were consistently high regardless of combined antibody status (Supplementary Table S7), indicating that antibody profiles did not substantially influence initial treatment response in this cohort. By contrast, when patients were stratified by combined anti-nephrin and anti-podocin antibody status, SDNS occurred not only in anti-nephrin–positive patients but also in anti-nephrin–negative/anti-podocin–positive patients, all of whom developed SDNS. Patients positive for both antibodies showed the highest incidence of SDNS, whereas the lowest incidence was observed in double-negative patients (Supplementary Table S7). These findings suggest that assessment of anti-podocin antibodies in addition to anti-nephrin antibodies may improve stratification of steroid-dependent disease beyond anti-nephrin antibody status alone; however, given the limited number of events, these analyses should be considered exploratory and hypothesis-generating. Prior large-scale studies also support a link between anti-nephrin antibodies and relapse-prone disease. In a cohort of nearly 600 patients with MCNS and primary FSGS, Yue Shu et al. found that anti-nephrin antibody positivity was associated with frequent relapses^15^. Given that frequent relapses often precede steroid dependence, their results are consistent with the possibility that anti-nephrin antibodies mark patients at risk for SDNS.

We also observed a higher proportion of males in the antibody-positive group. To our knowledge, sex-specific differences in anti-nephrin antibody positivity have not been reported previously. Although the biological significance of this finding remains uncertain, MN and FSGS are known to occur more frequently in males ^2,19^, whereas MCNS shows a male predominance in young children that disappears in adulthood ^20^. Moreover, sex-related differences in podocyte susceptibility and immune regulation have been described ^21,22^. Whether our observation reflects a true sex-associated immunological tendency or sampling variation requires confirmation in larger studies.

In conclusion, anti-nephrin antibodies were enriched in MCNS, whereas anti-podocin antibodies were detected across disease categories including MN. Antibody profiling—particularly combined assessment of anti-nephrin and anti-podocin antibodies—may help stratify disease severity and steroid dependence and serve as a marker of disease activity in adult-onset NS.

This study has several limitations. The FSGS subgroup was small and included patients with comorbidities suggestive of secondary/adaptive FSGS, and monogenic causes were not systematically assessed; therefore, some misclassification cannot be excluded. In addition, while routine biopsy immunofluorescence was reviewed, we did not perform systematic tissue-level analyses of nephrin/podocin localization or IgG co-localization using super-resolution imaging. Finally, although baseline anti-nephrin antibody positivity was associated with SDNS, the exploratory ROC analysis showed only modest discrimination and was constrained by the small sample size and a highly skewed distribution with many undetectable titers. Accordingly, our data do not support a clinically applicable cutoff, which will require validation in larger cohorts.

## Supplementary Information

Below is the link to the electronic supplementary material.

Supplementary Material 1

Supplementary Material 2

Supplementary Material 3

## Data Availability

The datasets generated and/or analyzed during the current study are available from the corresponding author on reasonable request.
