# Supplementary material for: Autoantibodies against nephrin and podocin are associated with disease severity and steroid dependence in adult-onset nephrotic syndrome
Source: Sci Rep. 2026 Mar 16;16:13724. doi: 10.1038/s41598-026-43612-7 (PMC13125207; doi:10.1038/s41598-026-43612-7)
Supplement: Supplementary file 1 — Supplementary Material 1 [file 41598_2026_43612_MOESM1_ESM.docx]

Supplementary Table S1. Individual serum anti-nephrin and anti-podocin antibody titers.

| Group | Age | Gender | Anti-Nephrin (AU/mL) | Anti-Podocin　(AU/mL) | Initial treatment regimen |  |  |
| --- | --- | --- | --- | --- | --- | --- | --- |
| MCNS | 22 | Male | 0.00 | 0.00 | IVMP+PSL+CsA |  |  |
| MCNS | 22 | Male | 216.98 | 1.66 | IVMP+PSL |  |  |
| MCNS | 63 | Male | 160.42 | 36.74 | IVMP+PSL+CsA |  |  |
| MCNS | 27 | Female | 646.55 | 4.61 | IVMP+PSL+CsA |  |  |
| MCNS | 68 | Male | 0.00 | 375.21 | CsA |  |  |
| MCNS | 40 | Male | 18.35 | 10.22 | IVMP+PSL+CsA |  |  |
| MCNS | 41 | Female | 0.00 | 2.11 | IVMP+PSL+CsA |  |  |
| MCNS | 66 | Female | 0.00 | 25.76 | IVMP+PSL+CsA |  |  |
| MCNS | 83 | Male | 0.00 | 0.00 | IVMP+PSL+CsA |  |  |
| MCNS | 58 | Female | 155.50 | 1.07 | IVMP+PSL+CsA |  |  |
| MCNS | 49 | Male | 0.00 | 0.00 | PSL+CsA |  |  |
| MCNS | 47 | Male | 0.00 | 0.00 | IVMP+PSL |  |  |
| MCNS | 73 | Female | 0.00 | 0.00 | IVMP+PSL+CsA |  |  |
| MCNS | 36 | Female | 0.00 | 31.64 | IVMP+PSL+CsA |  |  |
| MCNS | 31 | Female | 0.00 | 0.00 | No immunosuppressive therapy |  |  |
| MCNS | 75 | Female | 141.86 | 0.00 | IVMP+PSL+CsA |  |  |
| MCNS | 59 | Male | 41.13 | 80.46 | IVMP+PSL+CsA |  |  |
| MCNS | 40 | Female | 0.00 | 0.00 | PSL |  |  |
| MCNS | 27 | Female | 0.00 | 0.00 | PSL + CsA |  |  |
| MCNS | 85 | Female | 0.00 | 0.00 | PSL + CsA |  |  |
| MCNS | 29 | Female | 0.00 | 0.00 | No immunosuppressive therapy |  |  |
| MCNS | 70 | Female | 0.00 | 0.00 | PSL |  |  |
| MCNS | 29 | Female | 0.00 | 0.00 | IVMP+PSL |  |  |
| MCNS | 85 | Male | 92.72 | 0.00 | PSL |  |  |
| MCNS | 48 | Female | 0.00 | 4.73 | IVMP+PSL+CsA |  |  |
| MCNS | 65 | Male | 0.00 | 0.00 | IVMP+PSL+CsA |  |  |
| MCNS | 63 | Male | 26.48 | 304.06 | PSL + CsA |  |  |
| MCNS | 76 | Female | 285.58 | 1678.10 | PSL + CsA |  |  |
| MCNS | 39 | Male | 0.00 | 0.00 | IVMP+PSL+CsA |  |  |
| MCNS | 21 | Female | 0.00 | 3.49 | IVMP+PSL+CsA |  |  |
| MCNS | 34 | Male | 0.00 | 0.61 | IVMP+PSL |  |  |
| MCNS | 21 | Male | 0.00 | 0.00 | IVMP+PSL+CsA |  |  |
| MCNS | 59 | Female | 0.00 | 0.00 | IVMP+PSL+CsA |  |  |
| MCNS | 77 | Male | 0.00 | 577.32 | PSL + CsA |  |  |
| MCNS | 18 | Female | 34.91 | 159.09 | IVMP+PSL+CsA |  |  |
| MCNS | 74 | Female | 0.00 | 0.00 | IVMP+PSL+CsA |  |  |
| MCNS | 47 | Male | 0.00 | 0.00 | PSL |  |  |
| MCNS | 79 | Male | 102.93 | 0.00 | IVMP+PSL+CsA |  |  |
| MCNS | 63 | Female | 15.38 | 25.48 | PSL + CsA |  |  |
| MCNS | 62 | Male | 14.39 | 0.00 | IVMP+PSL+CsA |  |  |
| MCNS | 45 | Male | 71.99 | 9.53 | IVMP+PSL+CsA |  |  |
| MCNS | 18 | Male | 0.00 | 0.00 | IVMP+PSL+CsA |  |  |
| MCNS | 18 | Female | 0.00 | 6.56 | IVMP+PSL+CsA |  |  |
| MCNS | 63 | Female | 0.00 | 0.00 | IVMP+PSL+CsA |  |  |
| MCNS | 64 | Male | 90.11 | 23.06 | PSL + CsA |  |  |
| MCNS | 72 | Male | 216.37 | 57.21 | PSL |  |  |
| MCNS | 40 | Female | 27.74 | 0.00 | IVMP+PSL+CsA | Secondary cause of FSGS | Genetic testing |
| FSGS | 48 | Female | 0.00 | 23.89 | IVMP+PSL+CsA | None | Not performed |
| FSGS | 33 | Female | 0.00 | 0.00 | No immunosuppressive therapy | None | Not performed |
| FSGS | 29 | Male | 0.00 | 318.48 | No immunosuppressive therapy | None | Performed  (no pathogenic variant) |
| FSGS | 76 | Male | 0.00 | 0.00 | IVMP+PSL | None | Not performed |
| FSGS | 70 | Male | 0.00 | 0.00 | IVMP+PSL+CsA | None | Not performed |
| FSGS | 68 | Male | 158.66 | 38.88 | IVMP+PSL+CsA | Long-standing hypertension | Not performed |
| FSGS | 68 | Female | 0.00 | 0.00 | IVMP+PSL+CsA | Long-standing hypertension | Not performed |
| FSGS | 70 | Female | 0.00 | 0.00 | PSL + CsA | None | Not performed |
| FSGS | 37 | Male | 0.00 | 0.00 | No immunosuppressive therapy | Obesity | Not performed |
| FSGS | 27 | Female | 0.00 | 0.00 | IVMP+PSL+CsA | None | Not performed |
| FSGS | 80 | Female | 0.00 | 0.00 | No immunosuppressive therapy | Essential Thrombocythemia | Not performed |
| FSGS | 75 | Female | 0.00 | 0.00 | PSL + CsA | None | Not performed |
| FSGS | 75 | Male | 41.35 | 0.00 | IVMP+PSL+CsA | Long-standing hypertension | Not performed |
| FSGS | 57 | Female | 0.00 | 2.95 | IVMP+PSL+CsA | None | Not performed |
| PLA2R-MN | 56 | Male | 0.00 | 8.12 |  |  |  |
| PLA2R-MN | 75 | Female | 0.00 | 0.00 |  |  |  |
| PLA2R-MN | 66 | Female | 0.00 | 2.67 |  |  |  |
| PLA2R-MN | 52 | Male | 0.00 | 5.73 |  |  |  |
| PLA2R-MN | 59 | Male | 0.00 | 145.20 |  |  |  |
| PLA2R-MN | 64 | Male | 0.00 | 0.00 |  |  |  |
| PLA2R-MN | 64 | Male | 0.00 | 0.00 |  |  |  |
| PLA2R-MN | 53 | Female | 0.00 | 0.00 |  |  |  |
| PLA2R-MN | 67 | Male | 0.00 | 9.41 |  |  |  |
| PLA2R-MN | 63 | Female | 0.00 | 19.50 |  |  |  |
| PLA2R-MN | 56 | Female | 0.00 | 0.00 |  |  |  |
| PLA2R-MN | 59 | Male | 0.00 | 0.00 |  |  |  |
| PLA2R-MN | 79 | Male | 0.00 | 0.00 |  |  |  |
| PLA2R-MN | 73 | Male | 0.00 | 0.00 |  |  |  |
| PLA2R-MN | 67 | Male | 0.00 | 50.02 |  |  |  |
| PLA2R-MN | 58 | Male | 0.00 | 14.88 |  |  |  |
| PLA2R-MN | 75 | Male | 0.00 | 0.00 |  |  |  |
| PLA2R-MN | 64 | Male | 0.00 | 0.00 |  |  |  |
| PLA2R-MN | 65 | Male | 0.00 | 0.00 |  |  |  |
| PLA2R-MN | 71 | Female | 0.00 | 0.00 |  |  |  |
| PLA2R-MN | 74 | Female | 0.00 | 0.00 |  |  |  |
| PLA2R-MN | 72 | Female | 0.00 | 0.00 |  |  |  |
| PLA2R-MN | 54 | Male | 0.00 | 0.00 |  |  |  |
| PLA2R-MN | 76 | Male | 0.00 | 1553.52 |  |  |  |
| PLA2R-MN | 76 | Female | 0.00 | 0.00 |  |  |  |
| PLA2R-MN | 57 | Male | 0.00 | 2.67 |  |  |  |
| PLA2R-MN | 53 | Female | 0.00 | 0.00 |  |  |  |
| PLA2R-MN | 71 | Male | 0.00 | 0.00 |  |  |  |
| PLA2R-MN | 63 | Male | 0.00 | 60.40 |  |  |  |
| PLA2R-MN | 60 | Female | 0.00 | 3.49 |  |  |  |
| PLA2R-MN | 72 | Male | 0.00 | 0.00 |  |  |  |
| PLA2R-MN | 66 | Female | 0.00 | 4.78 |  |  |  |
| PLA2R-MN | 62 | Female | 0.00 | 14.97 |  |  |  |
| PLA2R-MN | 78 | Female | 0.00 | 0.00 |  |  |  |
| PLA2R-MN | 47 | Male | 0.00 | 0.00 |  |  |  |
| PLA2R-MN | 71 | Male | 0.00 | 15.40 |  |  |  |
| PLA2R-MN | 46 | Female | 0.00 | 0.00 |  |  |  |
| PLA2R-MN | 55 | Male | 0.00 | 0.00 |  |  |  |
| PLA2R-MN | 66 | Female | 362.64 | 284.90 |  |  |  |
| PLA2R-MN | 60 | Male | 0.00 | 0.00 |  |  |  |
| NELL1-MN | 71 | Male | 0.00 | 0.00 |  |  |  |
| NELL1-MN | 81 | Male | 0.00 | 229.65 |  |  |  |
| NELL1-MN | 85 | Male | 0.00 | 71.45 |  |  |  |
| NELL1-MN | 61 | Female | 0.00 | 80.10 |  |  |  |
| NELL1-MN | 73 | Male | 0.00 | 24.17 |  |  |  |
| NELL1-MN | 61 | Male | 0.00 | 0.00 |  |  |  |
| NELL1-MN | 70 | Male | 0.00 | 252.23 |  |  |  |
| NELL1-MN | 33 | Female | 0.00 | 188.86 |  |  |  |
| NELL1-MN | 68 | Male | 0.00 | 0.00 |  |  |  |
| NELL1-MN | 80 | Female | 0.00 | 0.00 |  |  |  |
| NELL1-MN | 81 | Female | 26.97 | 4.25 |  |  |  |
| NELL1-MN | 68 | Female | 0.00 | 0.00 |  |  |  |
| NELL1-MN | 68 | Male | 0.00 | 3293.31 |  |  |  |
| Control | 49 | Male | 0.00 | 2.60 |  |  |  |
| Control | 55 | Female | 0.00 | 0.00 |  |  |  |
| Control | 31 | Female | 0.00 | 0.00 |  |  |  |
| Control | 72 | Male | 0.00 | 47.06 |  |  |  |
| Control | 39 | Male | 0.15 | 0.00 |  |  |  |
| Control | 38 | Male | 0.00 | 2.52 |  |  |  |
| Control | 62 | Female | 0.00 | 0.00 |  |  |  |
| Control | 30 | Female | 0.00 | 19.35 |  |  |  |
| Control | 31 | Female | 0.00 | 0.00 |  |  |  |
| Control | 41 | Male | 0.00 | 10.70 |  |  |  |
| Control | 35 | Female | 0.00 | 6.48 |  |  |  |
| Control | 38 | Male | 0.00 | 44.24 |  |  |  |
| Control | 39 | Male | 0.00 | 0.00 |  |  |  |
| Control | 51 | Female | 0.00 | 10.19 |  |  |  |
| Control | 45 | Female | 0.00 | 86.42 |  |  |  |
| Control | 28 | Male | 0.00 | 0.00 |  |  |  |
| Control | 36 | Female | 0.00 | 9.77 |  |  |  |
| Control | 40 | Male | 0.00 | 31.15 |  |  |  |
| Control | 33 | Female | 0.00 | 0.00 |  |  |  |
| Control | 28 | Male | 0.00 | 1.19 |  |  |  |
| Control | 29 | Female | 0.00 | 49.86 |  |  |  |
| Control | 25 | Male | 0.00 | 10.18 |  |  |  |
| Control | 25 | Male | 0.00 | 6.08 |  |  |  |
| Control | 26 | Male | 0.00 | 0.00 |  |  |  |
| Control | 35 | Male | 0.00 | 12.49 |  |  |  |
| Control | 36 | Male | 0.00 | 50.85 |  |  |  |
| Control | 24 | Male | 0.00 | 19.06 |  |  |  |
| Control | 25 | Male | 0.00 | 33.22 |  |  |  |
| Control | 25 | Male | 0.00 | 25.31 |  |  |  |
| Control | 41 | Female | 0.00 | 25.26 |  |  |  |
| Control | 45 | Male | 0.00 | 56.79 |  |  |  |
| Control | 32 | Male | 0.00 | 32.23 |  |  |  |
| Control | 30 | Male | 0.00 | 42.93 |  |  |  |
| Control | 53 | Male | 0.00 | 89.61 |  |  |  |
| Control | 51 | Male | 0.00 | 64.06 |  |  |  |
| Control | 30 | Male | 0.00 | 0.00 |  |  |  |
| Control | 45 | Male | 0.00 | 0.00 |  |  |  |
| Control | 31 | Male | 0.00 | 0.00 |  |  |  |
| Control | 29 | Male | 0.00 | 57.09 |  |  |  |
| Control | 30 | Male | 0.00 | 45.15 |  |  |  |

Abbreviations: IVMP, intravenous methylprednisolone pulse therapy; PSL, oral prednisolone; CsA, cyclosporine A
