# Supplementary material for: Autoantibodies against nephrin and podocin are associated with disease severity and steroid dependence in adult-onset nephrotic syndrome
Source: Sci Rep. 2026 Mar 16;16:13724. doi: 10.1038/s41598-026-43612-7 (PMC13125207; doi:10.1038/s41598-026-43612-7)
Supplement: Supplementary file 2 — Supplementary Material 2 [file 41598_2026_43612_MOESM2_ESM.docx]

Supplementary Table S2. ELISA assay quality control and reproducibility

|  | Intra-assay CV | Inter-assay CV |
| --- | --- | --- |
| Anti-nephrin antibodies | 3.7% [1.3-6.8] | 19.4% [16.6-22.6] |
| Anti-podocin antibodies | 2.9% [1.3-7.5] | 22.9% [13.4-28.7] |

Intra-assay coefficients of variation (CVs) were calculated from duplicate measurements within the same assay run and are presented as median (interquartile range). Inter-assay CVs were calculated from standard curve measurements across independent assay runs and are presented as median (interquartile range).

Supplementary Table S3. Sensitivity analysis of anti-nephrin antibody positivity rates using alternative cutoff definitions

Anti-nephrin antibodies

|  | Study cutoff | Maximum | 95th  percentile | 97.5th  percentile | 99th  percentile |
| --- | --- | --- | --- | --- | --- |
| Cutoff value | 0.2 | 0.15 | 0 | 0.003 | 0.09 |
| MCNS | 18/47 (38.3%) | 18/47 (38.3%) | 47/47 (100.0%) | 18/47 (38.3%) | 18/47 (38.3%) |
| FSGS | 2/14 (14.3%) | 2/14 (14.3%) | 14/14 (100.0%) | 2/14 (14.3%) | 2/14 (14.3%) |
| PLA2R-MN | 1/40 (2.5%) | 1/40 (2.5%) | 40/40 (100.0%) | 1/40 (2.5%) | 1/40 (2.5%) |
| NELL1-MN | 1/13 (7.7%) | 1/13 (7.7%) | 13/13 (100.0%) | 1/13 (7.7%) | 1/13 (7.7%) |

Antibody positivity rates are shown for each disease group using different cutoff definitions for anti-nephrin antibodies. The study cutoff was defined as an anti-nephrin antibody titer ≥0.2 AU/mL.

Alternative cutoffs were derived from healthy controls, including the maximum value, 95th percentile, 97.5th percentile, and 99th percentile. Positivity rates are expressed as the number of positive patients divided by the total number of patients in each group (%).

Supplementary Table S4. Sensitivity analysis of anti-podocin antibody positivity rates using alternative cutoff definitions

Anti-podocin antibodies

|  | Study cutoff | Maximum | 95th  percentile | 97.5th  percentile | 99th  percentile |
| --- | --- | --- | --- | --- | --- |
| Cutoff value | 90 | 89.6 | 65.2 | 86.5 | 88.4 |
| MCNS | 5/47 (10.6%) | 5/47 (10.6%) | 6/47 (12.8%) | 5/47 (10.6%) | 5/47 (10.6%) |
| FSGS | 1/14 (7.1%) | 1/14 (7.1%) | 1/14 (7.1%) | 1/14 (7.1%) | 1/14 (7.1%) |
| PLA2R-MN | 3/40 (7.5%) | 3/40 (7.5%) | 3/40 (7.5%) | 3/40 (7.5%) | 3/40 (7.5%) |
| NELL1-MN | 4/13 (30.8%) | 4/13 (30.8%) | 6/13 (46.2%) | 4/13 (30.8%) | 4/13 (30.8%) |

Antibody positivity rates are shown for each disease group using different cutoff definitions for anti-podocin antibodies. The study cutoff was defined as an anti-podocin antibody titer ≥90 AU/mL.

Alternative cutoffs were derived from healthy controls, including the maximum value, 95th percentile, 97.5th percentile, and 99th percentile. Positivity rates are expressed as the number of positive patients divided by the total number of patients in each group (%).

MCNS, minimal change nephrotic syndrome; FSGS, focal segmental glomerulosclerosis; PLA2R-MN, phospholipase A2 receptor–associated membranous nephropathy; NELL1-MN, neural epidermal growth factor–like 1–associated membranous nephropathy.

Supplementary Table S5. Baseline clinical characteristics of patients with MCNS and FSGS stratified by anti-nephrin antibody status, excluding anti-podocin–only positive cases

|  | **Total (n=58)** | **Anti-nephrin**  **Positive (n=20)** | **Anti-nephrin**  **Negative (n=38)** | ***p* value** |
| --- | --- | --- | --- | --- |
| **Male/Female** | 26 (44.8%) / 32 (55.2%) | 13 (65.0%) / 7 (35.0%) | 13 (34.2%) / 25 (65.8%) | 0.03* |
| **Age (years)** | 57.50 [34.50–70.00] | 63.00 [43.75–72.75] | 47.50 [31.50–69.50] | 0.211 |
| **anti-Nephrin titer (AU/mL)** | 0.00 [0.00–33.12] | 91.41 [33.12–159.10] | 0.00 [0.00–0.00] | <0.001* |
| **anti-Podocin titer (AU/mL)** | 0.00 [0.00–6.10] | 9.88 [0.00–43.46] | 0.00 [0.00–0.00] | <0.001* |
| **Cre (mg/dL)** | 0.99 [0.73–1.22] | 1.03 [0.92–1.45] | 0.89 [0.64–1.11] | 0.071 |
| **eGFR (mL/min/1.73 m²)** | 62.70 [41.55–79.50] | 59.70 [38.55–66.20] | 66.10 [45.55–84.50] | 0.098 |
| **TP (g/dL)** | 4.80 [4.40–5.55] | 4.70 [4.38–5.20] | 5.00 [4.40–5.75] | 0.64 |
| **Alb (g/dL)** | 1.85 [1.22–2.10] | 1.50 [1.28–1.90] | 2.00 [1.22–2.20] | 0.138 |
| **IgG (mg/dL)** | 733.50 [505.25–1001.50] | 783.00 [568.00–930.75] | 696.50 [505.25–1014.75] | 0.832 |
| **IgE (IU/mL)** | 321.50 [76.28–1727.75] | 233.50 [82.50–1490.00] | 504.50 [81.42–1663.25] | 0.83 |
| **CH50 (U/mL)** | 53.20 [46.20–59.25] | 54.60 [50.50–60.00] | 52.70 [42.80–57.50] | 0.14 |
| **C3 (mg/dL)** | 131.50 [115.50–151.25] | 142.00 [128.50–164.00] | 129.00 [114.00–149.00] | 0.032* |
| **C4 (mg/dL)** | 36.50 [29.00–46.50] | 40.00 [32.00–48.50] | 33.00 [29.00–42.00] | 0.051 |
| **ANA positive / negative** | 19 (33.3%) / 38 (66.7%) | 7 (35.0%) / 13 (65.0%) | 12 (32.4%) / 25 (67.6%) | 1.000 |
| **uPCR (g/gCr)** | 11.30 [7.99–14.48] | 12.73 [10.85–17.29] | 10.26 [6.14–13.94] | 0.02* |
| **Selectivity Index** | 0.13 [0.09–0.19] | 0.12 [0.10–0.24] | 0.14 [0.08–0.18] | 0.575 |
| **Urinary β2MG (µg/L)** | 287.00 [111.00–688.00] | 291.00 [161.75–619.00] | 282.00 [111.00–752.00] | 0.902 |

Patients who were positive only for anti-podocin antibodies (MCNS, n = 2; FSGS, n = 1) were excluded from this analysis.

Values are number (%) and median [IQR, interquartile range].

Abbreviations: Cre, serum creatinine; eGFR, estimated glomerular filtration rate; TP, total protein; Alb, serum albumin; ANA, antinuclear antibody; uPCR, urine protein-to-creatinine ratio; β2MG, urinary β2-microglobulin.

Supplementary Table S6. Baseline clinical characteristics of anti-nephrin-negative patients stratified by anti-podocin antibody positivity.

|  | **Total (n=92)** | **Anti-podocin**  **Positive (n=9)** | **Anti-podocin**  **Negative (n=83)** | ***p* value** |
| --- | --- | --- | --- | --- |
| **Male/Female** | 48 (52.2%) / 44 (47.8%) | 8 (88.9%) / 1 (11.1%) | 40 (48.2%) / 43 (51.8%) | 0.032* |
| **Age (years)** | 63.00 [47.75–71.00] | 68.00 [59.00–76.00] | 63.00 [47.50–71.00] | 0.311 |
| **anti-Nephrin titer (AU/mL)** | 0.00 [0.00–0.00] | 0.00 [0.00–0.00] | 0.00 [0.00–0.00] | NA |
| **anti-Podocin titer (AU/mL)** | 0.00 [0.00–6.95] | 318.48 [229.65–577.32] | 0.00 [0.00–2.81] | <0.001* |
| **Cre (mg/dL)** | 0.87 [0.67–1.05] | 1.00 [0.88–1.50] | 0.80 [0.67–1.03] | 0.052 |
| **eGFR (mL/min/1.73 m²)** | 64.95 [51.01–79.25] | 57.60 [35.27–69.23] | 65.10 [53.90–79.50] | 0.198 |
| **TP (g/dL)** | 5.10 [4.60–6.00] | 5.70 [4.80–6.00] | 5.10 [4.60–6.00] | 0.502 |
| **Alb (g/dL)** | 2.20 [1.80–2.92] | 2.30 [1.60–2.90] | 2.20 [1.80–2.95] | 0.880 |
| **uPCR (g/gCr)** | 8.55 [3.71–12.38] | 7.82 [3.70–11.33] | 8.68 [3.75–12.45] | 0.849 |

The anti-nephrin–negative cohort included patients with MCNS, FSGS, PLA2R-MN, and NELL1-MN.

Values are number (%) and median [IQR, interquartile range].

Abbreviations: Cre, serum creatinine; eGFR, estimated glomerular filtration rate; TP, total protein; Alb, serum albumin; uPCR, urine protein-to-creatinine ratio.

Supplementary Table S7. Incidence of remissions and steroid-dependent nephrotic syndrome according to combined anti-nephrin and anti-podocin antibody status.

| **Antibody Profile** | **JSN-ICR2** | **JSN-ICR1** | **JSN-CR** |
| --- | --- | --- | --- |
| Nephrin− / Podocin− | 30/33 (90.9%) | 30/33 (90.9%) | 24/33 (72.7%) |
| Nephrin− / Podocin+ | 2/2 (100.0%) | 2/2 (100.0%) | 2/2 (100.0%) |
| Nephrin+ / Podocin− | 17/17 (100.0%) | 17/17 (100.0%) | 15/17 (88.2%) |
| Nephrin+ / Podocin+ | 3/3 (100.0%) | 3/3 (100.0%) | 2/3 (66.7%) |

| **Antibody Profile** | **KDIGO-PR** | **KDIGO-CR** | **SDNS** |
| --- | --- | --- | --- |
| Nephrin− / Podocin− | 30/33 (90.9%) | 29/33 (87.9%) | 11/26 (42.3%) |
| Nephrin− / Podocin+ | 2/2 (100.0%) | 2/2 (100.0%) | 2/2 (100.0%) |
| Nephrin+ / Podocin− | 17/17 (100.0%) | 15/17 (88.2%) | 7/11 (63.6%) |
| Nephrin+ / Podocin+ | 3/3 (100.0%) | 3/3 (100.0%) | 3/3 (100.0%) |

Remission rates according to both JSN and KDIGO criteria were uniformly high across all antibody profiles. In contrast, the incidence of SDNS appeared numerically higher in patients with anti-nephrin positivity, particularly in those with combined anti-nephrin and anti-podocin antibodies

Abbreviations: JSN, Japanese Society of Nephrology; ICR, incomplete remission; CR, complete remission; PR, partial remission; KDIGO, Kidney Disease: Improving Global Outcomes; SDNS, steroid-dependent nephrotic syndrome.

Supplementary Figure S2

Receiver operating characteristic (ROC) curve of baseline anti-nephrin antibody titers for predicting subsequent development of steroid-dependent nephrotic syndrome (SDNS). The area under the curve (AUC) was 0.63 (95% CI, 0.48–0.78). The optimal threshold based on the Youden index showed moderate specificity but limited sensitivity.
