# Supplementary figures and images for: Autoantibodies against nephrin and podocin are associated with disease severity and steroid dependence in adult-onset nephrotic syndrome
Source: Sci Rep. 2026 Mar 16;16:13724. doi: 10.1038/s41598-026-43612-7 (PMC13125207; doi:10.1038/s41598-026-43612-7)

Supplemental Figure S1A

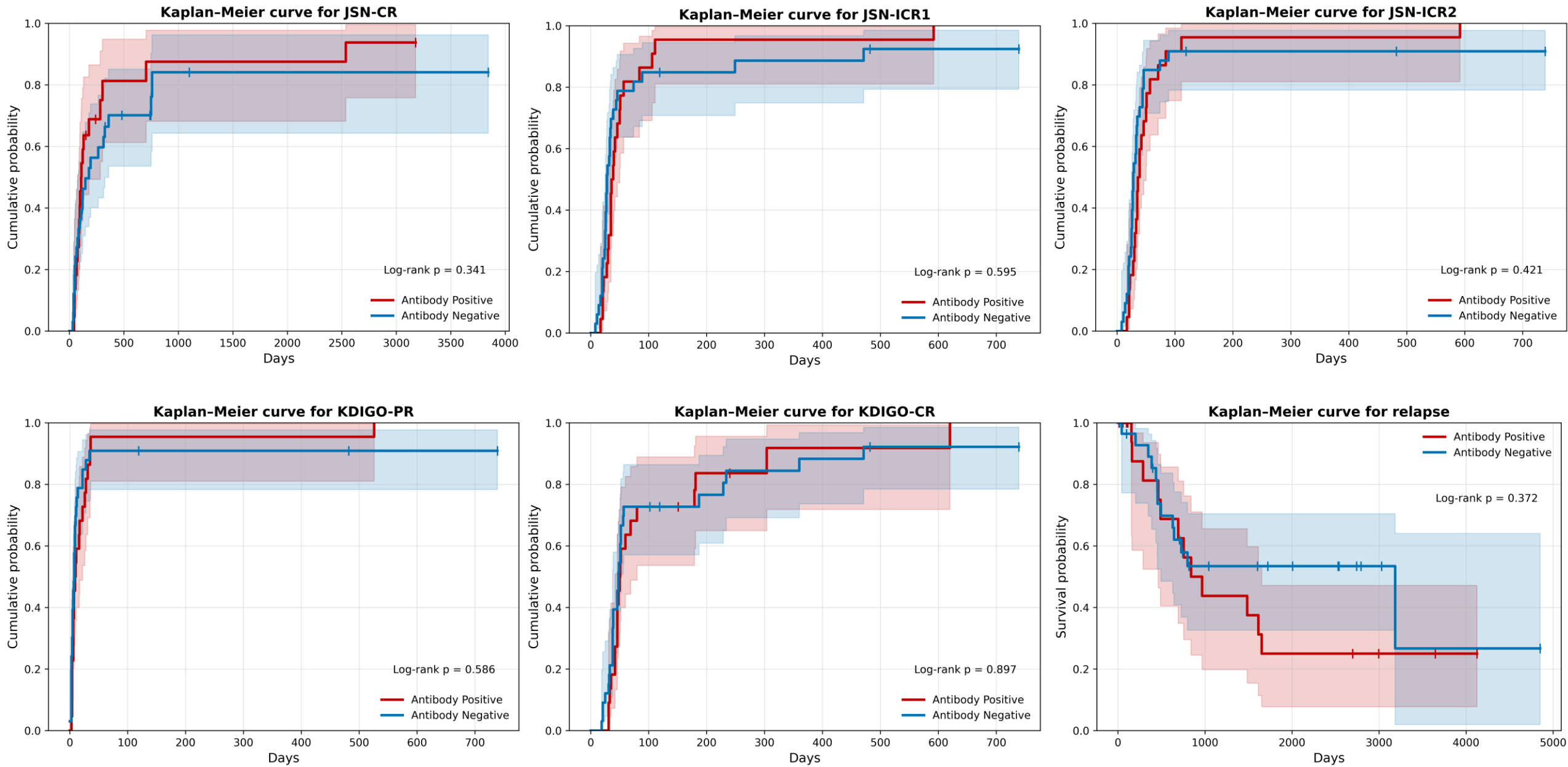

Supplemental Figure S1B

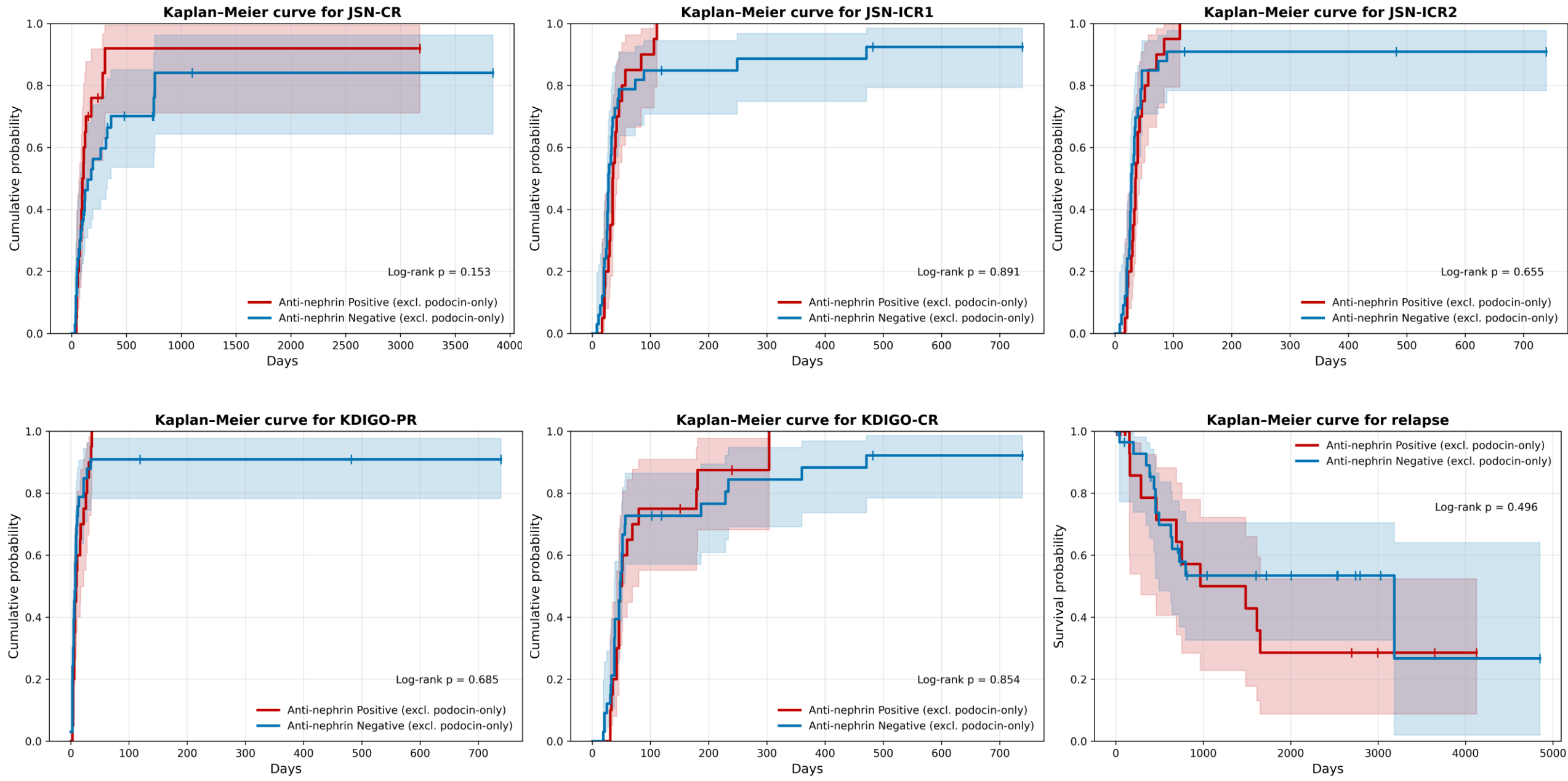

Supplement: Supplementary file 3 — Supplementary Material 3 [file 41598_2026_43612_MOESM3_ESM.pdf]
